# Correspondence on adverse conditions of COVID-19 vaccination

**DOI:** 10.1186/s43162-023-00211-6

**Published:** 2023-04-24

**Authors:** Amnuay Kleebayoon, Viroj Wiwanitkit

**Affiliations:** 1 Private Academic Consultant, Samraong, Cambodia; 2 Chandigarh University, Mohali, Punjab India; 3 Joesph Ayobabalola University, Ikeji-Arakeji, Nigeria

Dear Editor,

We would like to comment on the article *An analysis of fatal adverse conditions in temporal association of COVID-19 vaccination to boost the safety of vaccination for COVID-19* [1]. Muslim et al. reviewed the literature and collected data from 15 studies, totaling 22 cases of fatal adverse condition/death associated with COVID-19 vaccination [1]. According to Muslim et al., an analysis of these data shows that many of those who died (40.90%) were previously healthy people. Within 3 weeks of vaccination, all of those who died developed symptoms or were admitted to the hospital. 86.36% of deaths occurred within 3 weeks of vaccination/presentation/admission/intervention [1]. Muslim et al. concluded that knowledge of fatal adverse conditions in the temporal association of vaccination will aid in dealing with these situations and further improving the safety of the vaccination drive [1].

We both concur that there may be a sizable fatality rate associated with cerebral venous sinus thrombosis brought on by the COVID-19 vaccination. A fascinating clinical problem is the unfavorable reaction to COVID-19 immunization. Although case-specific data can be included in articles, there is no way to prove conclusively how confounding variables affect the results [2]. Finding the right answer could be challenging. The current research reads like an investigation of the impacts of vaccine aversion and has a very subjective appearance. Finding the precise reason for a vaccination reaction may be challenging due to a paucity of clinical data describing the physiological and immunological status of COVID-19 vaccine recipients prior to vaccine injection. Even when they do exist, comorbidities are rarely mentioned in clinical records. Determining the precise patho-immuno-pharmacological link can be difficult at times due to a lack of expertise. It may be difficult to comprehend how concurrent medical issues influence clinical outcomes [3]. The genetic influences are the least important [4]. More specifics are difficult to provide when none of the current report’s conclusions is supported by new data.
